# Aerodynamic characteristics in upper airways among orthodontic patients and its association with adenoid nasopharyngeal ratios in lateral cephalograms

**DOI:** 10.1186/s12880-021-00659-4

**Published:** 2021-08-23

**Authors:** Xin Feng, Yicheng Chen, Weihua Cai, Stein Atle Lie, Kristina Hellén-Halme, Xie-Qi Shi

**Affiliations:** 1 Department of Clinical Dentistry, Faculty of Medicine, University of Bergen, Årstadveien 19, 5009 Bergen, Norway; 2 School of Energy Science and Engineering, Harbin Institute of Technology, Xi Da Zhi Street, Nangang, Harbin, 150001 People’s Republic of China; 3 School of Energy and Power Engineering, Northeast Electric Power University, Changchun Road 169, Changchun, 132012 People’s Republic of China; 4 Department of Oral and Maxillofacial Radiology, Faculty of Odontology, Malmö University, 205 06, Malmö, Sweden

**Keywords:** Lateral cephalogram, CBCT, Adenoids, Upper airway, Computational fluid dynamics

## Abstract

**Background:**

Adenoid hypertrophy among orthodontic patients may be detected in lateral cephalograms. The study investigates the aerodynamic characteristics within the upper airway (UA) by means of computational fluid dynamics (CFD) simulation. Furthermore, airflow features are compared between subgroups according to the adenoidal nasopharyngeal (AN) ratios.

**Methods:**

This retrospective study included thirty-five patients aged 9–15 years having both lateral cephalogram and cone beam computed tomography (CBCT) imaging that covered the UA region. The cases were divided into two subgroups according to the AN ratios measured on the lateral cephalograms: Group 1 with an AN ratio < 0.6 and Group 2 with an AN ratio ≥ 0.6. Based on the CBCT images, segmented UA models were created and the aerodynamic characteristics at inspiration and expiration were simulated by the CFD method for the two groups. The studied aerodynamic parameters were pressure drop (ΔP), maximum midsagittal velocity (V_ms_), maximum wall shear stress (P_ws_), and minimum wall static pressure (P_w_).

**Results:**

The maximum V_ms_ exhibits nearly 30% increases in Group 2 at both inspiration (*p* = 0.013) and expiration (*p* = 0.045) compared to Group 1. For the other aerodynamic parameters such as ΔP, the maximum P_ws_, and minimum P_w_, no significant difference is found between the two groups.

**Conclusions:**

The maximum V_ms_ seems to be the most sensitive aerodynamic parameter for the groups of cases. An AN ratio of more than 0.6 measured on a lateral cephalogram may associate with a noticeably increased maximum V_ms_, which could assist clinicians in estimating the airflow features in the UA.

## Background

Hans Wilhelm Meyer first described the clinical condition of nasal obstruction caused by adenoid hypertrophy (AH) in 1868 [1]. Recurrent or chronic upper airway (UA) infections, allergic inflammation, and immune response may lead to AH, one of the most common causes of UA obstruction in children and adolescents [2, 3]. Many studies have suggested that AH is related to cardiopulmonary complications, craniofacial growth, and obstructive sleep apnea [4–6]. The presence of AH causes a varying degree of nasopharyngeal obstruction, mouth breathing, snoring, and disturbance in craniofacial growth. A specific “adenoid face” is characteristic among AH patients with a narrow and high maxillary arch, abnormal position of the tongue, and retrusion of the mandible [7]. Thus, early identification of the airflow alteration caused by AH in orthodontic patients is essential to avoid further complications. Currently, the nasendoscopy is considered the standard for clinically assessing the adenoid size on cooperative children [8].

Lateral cephalograms, cone beam computed tomography (CBCT), computed tomography (CT), and magnetic resonance imaging have been investigated to evaluate the size, shape, and location of the adenoid [9–12]. Among the radiological modalities, the lateral cephalogram has been widely applied in children and adolescents to depict and trace skeletal structures and occlusion during the orthodontic treatment process. Since the presence of AH affects occlusion and craniofacial morphology [7, 13], adenoid assessment is an integral part of cephalometric analysis for this group of children.

The adenoid size and patency of the surrounding nasopharynx could be presented in terms of the absolute dimensions of adenoid thickness and nasopharyngeal width [14], percentage of adenoid-nasopharyngeal obstruction [10, 15], cross-sectional areas [12], volumes [16] of the adenoid and nasopharynx as well as the adenoidal nasopharyngeal (AN) ratio [9]. The AN ratio was found to correlate significantly with the nasopharyngeal volume [17], clinical endoscopic examination [18], and symptoms of obstructive sleeping [19]. Feng et al. [17] investigated the AN ratios in relation to the 3D volumetric data and recommended using the AN ratio as an initial screening method to estimate the nasopharyngeal volumes of patients younger than 15 years old. An AN ratio of 0.6 is considered a threshold when suspecting AH, whereas an AN ratio value of more than 0.7 has been well accepted for indicating pathological AH, and adenoidectomy may be suggested by clinicians after clinical assessment [20, 21]. The current diagnosis of AH is based on adenoid morphology; the ultimate effect of adenoid size on respiratory function in terms of airflow alteration is yet unclear.

Computational fluid dynamics (CFD) simulations may be the solution to link the UA morphology and airflow characteristics. CFD simulation is a well-established method for simulating the flow of gases or fluids and their interactions with the surrounding surfaces, as defined by boundary conditions. It has been widely used in the industry to predict the dynamic characteristics of the targeted flow. However, the application of CFD in dentistry was nevertheless sparse and had mainly been applied in evaluating the outcome of mandibular advancement devices in sleep-disordered breathing [22–24]. CFD has been accepted as an accurate and reliable method for associating the maxillofacial morphology and the UA’s aerodynamic characteristics [22, 25, 26].

The study aims to investigate the aerodynamic characteristics within UA among orthodontic patients by CFD simulation. Furthermore, airflow features are compared between subgroups classified according to the AN ratios.

## Methods

### Sample size estimation

Maximum midsagittal velocity of the airflow in the UA is considered to be the primary outcome variable based on a previous study by Feng et al. [27]. A sample size of 30 will be needed to ensure an 80% power to reject the null hypothesis at a significance level of 5%, assuming differences in maximum midsagittal velocity and its standard deviation is 0.7 m/s and 0.5 m/s between the cases with an AN ratio < 0.6 and ≥ 0.6, and the ratio between the two groups is 2:1.

### Samples collection

This cross-sectional study is a subset of a longitudinal prospective study performed at Dalian Stomatological Hospital between 2015 and 2017, in which 2D and 3D images were compared for tracing anatomic landmarks before and after orthodontic treatment. The study was approved by the regional ethics review boards in Dalian, China (DLKQLL201604) and Bergen, Norway (2018/1547 REK Vest). Informed consent was obtained from all patients or their legal guardians. The baseline images from 2015 were retrospectively collected and employed in the current study. The inclusion criteria were individuals aged 9 to 15 years who had had both a lateral cephalogram and CBCT scan examined within one week. For CBCT images, the field of view was required to cover the UA regions, including the nasal cavity, nasopharynx, and oropharynx. The exclusion criteria were severe maxillofacial abnormalities and previous surgery on skeletal and soft tissue related to respiration. In the present study, ninety-two cases were initially included. X.F previewed all the CBCT scans and lateral cephalograms. Fifty-seven cases were excluded, of which 53 did not cover the UA, 3 scans had motion artefact and 1 showed suboptimal patient positioning. Eventually, thirty-five cases were recruited. All the cases were divided into two groups: Group 1 with AN ratios < 0.6 (n = 25) and Group 2 with AN ratios ≥ 0.6 (n = 10).

### Lateral cephalogram

**Fig. 1:**
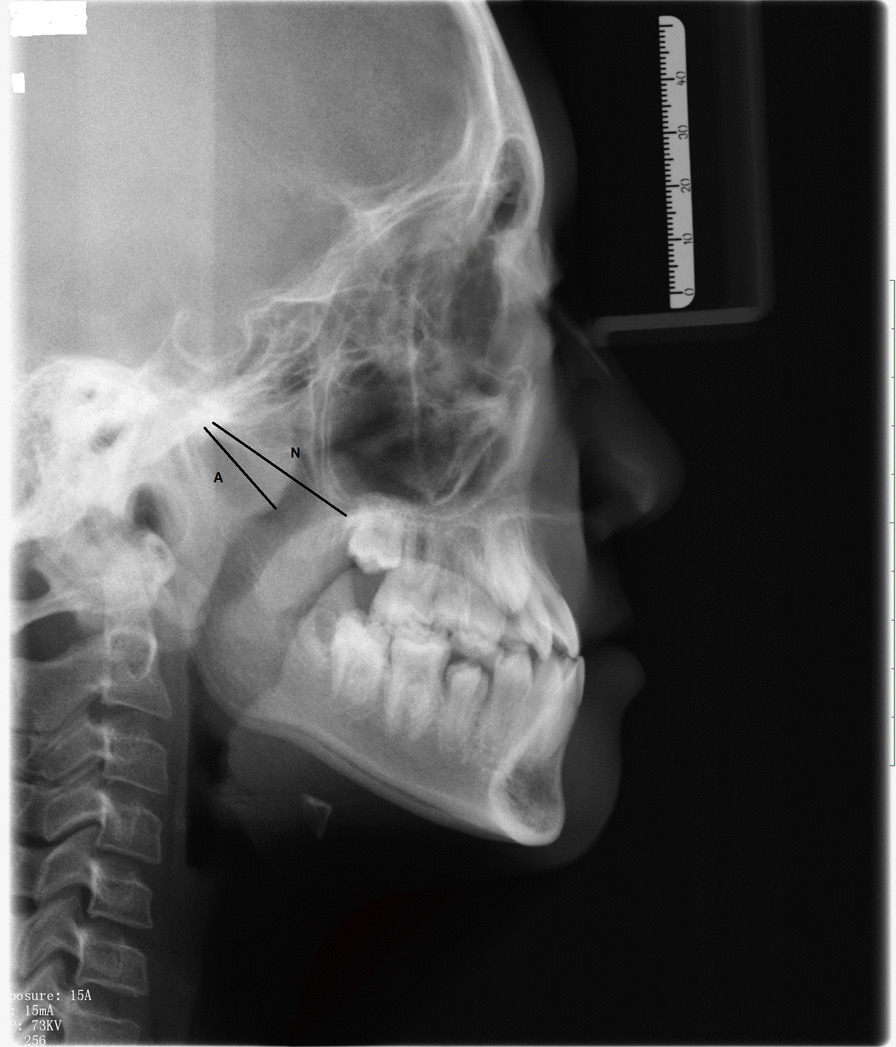
Calculating the adenoidal nasopharyngeal (AN) ratio on a lateral cephalogram. **A,** Perpendicular distance between maximum convexity of the adenoid shadow and the anterior margin of the basiocciput. **N,** Distance between the posterosuperior edge of the hard palate and the anteroinferior edge of the spheno-occipital synchondrosis

The AN ratios were measured and calculated on the lateral cephalograms captured by a digital pan/ceph system (ORTHOPHOS XG 5; Sirona Dental Systems, Bensheim, Germany) at 73 kVp and 15 mA with exposure times of 9.4 s and a contrast resolution of 16-bit depth. A is defined as a perpendicular distance between the point of maximal convexity of the adenoid to the anterior margin of the basiocciput. N is the distance between the posterosuperior edge of the hard palate and the anteroinferior edge of the spheno-occipital synchondrosis [9] (Fig. 1).

### CBCT scans

All CBCT scans were obtained by 3D eXam (KaVo, Biberach an der Riss, Germany). The following parameters were used: a field of view (FOV) of 16 × 13 cm, tube voltage of 120 kV, tube current of 5 mA, scanning time of 14.7 s, voxel size of 0.2 mm, and contrast resolution of 14-bit depth.

### CFD simulation

The CBCT images were imported in the digital imaging and communications in medicine (DICOM) format to MIMICS software (MIMICS, Materialise, Belgium) for later analysis. 3D renderings of the CBCT scans were oriented with axial planes parallel to the Frankfurt horizontal plane; the midsagittal planes intersected the nasion and anterior nasal spine, and the coronal plane was adjusted to the level of the porions. For each case, a mask was reconstructed, making sure the integrity of UA was displayed correctly. CFD simulation was then conducted on the 3D model within the mask region. The superior boundary of the studied UA was defined as a vertical plane in the nasal cavity, passing through the most posterior point of the middle turbinate, whereas the inferior boundary was a horizontal plane, in the pharynx, in line with the most anterior-inferior point of cervical vertebra 4. Each end of the boundary was extended by 20 mm to avoid flow reversing during the simulating process. The inlet and outlet of UA were set on the extended planes. A surface model was then created according to the extended 3D model for mesh generation. We chose tetrahedral and prismatic cells to construct the main body and boundary layer of the UA mesh (ANSYS, Inc., Canonsburg, Pennsylvania). The SST κ-ω model was used to calculate the aerodynamic characteristics of UA by applying ANSYS Fluent (ANSYS, Inc., Canonsburg, Pennsylvania). The wall of the UA was defined as no-slip, stationary, and rigid. The temperature and density of air were set as fixed. At inspiration, the inlet was set with the pressure 0 Pa and the outlet at a flow rate of -200 mL/s [28]. The corresponding values were -200 mL/s and 0 Pa at inlet and outlet at expiration.

The aerodynamic parameters applied and computed are listed in Table 1. The pressure drop (ΔP) refers to the pressure difference between a vertical plane through the most posterior point of the middle turbinate and a horizontal plane through the tip of the epiglottis.

**Table 1:** Description of the aerodynamic parameters evaluated applying the CFD simulation

Name	Unit	Definition
Maximum V_ms_	m/s	The maximum velocity on the midsagittal plane
ΔP	Pa	The pressure drop of airflow between the defined two planes
Maximum P_ws_	Pa	The maximum lateral pressure of airflow acting on the UA wall
Minimum P_w_	Pa	The minimum vertical pressure of airflow acting on the UA wall

Two experienced operators performed CFD simulations, one on all cases and one on ten randomly selected cases. The first operator repeated the measurements one month later on the ten selected cases.

### Data analyses

Data were processed using IBM-SPSS, version 25.0 (IBM, New York, NY, USA). Significance was set at p-values less than 0.05. The assumption of normal distribution for all variables was tested. An independent-samples T-test or Mann–Whitney U test was applied to compare the aerodynamic parameters between subgroups and between genders. The Intraclass Correlation Coefficient (ICC) was applied to test intra- and inter-observer reliability on the selected ten cases using a random number generator.

## Results

**Fig. 2:**
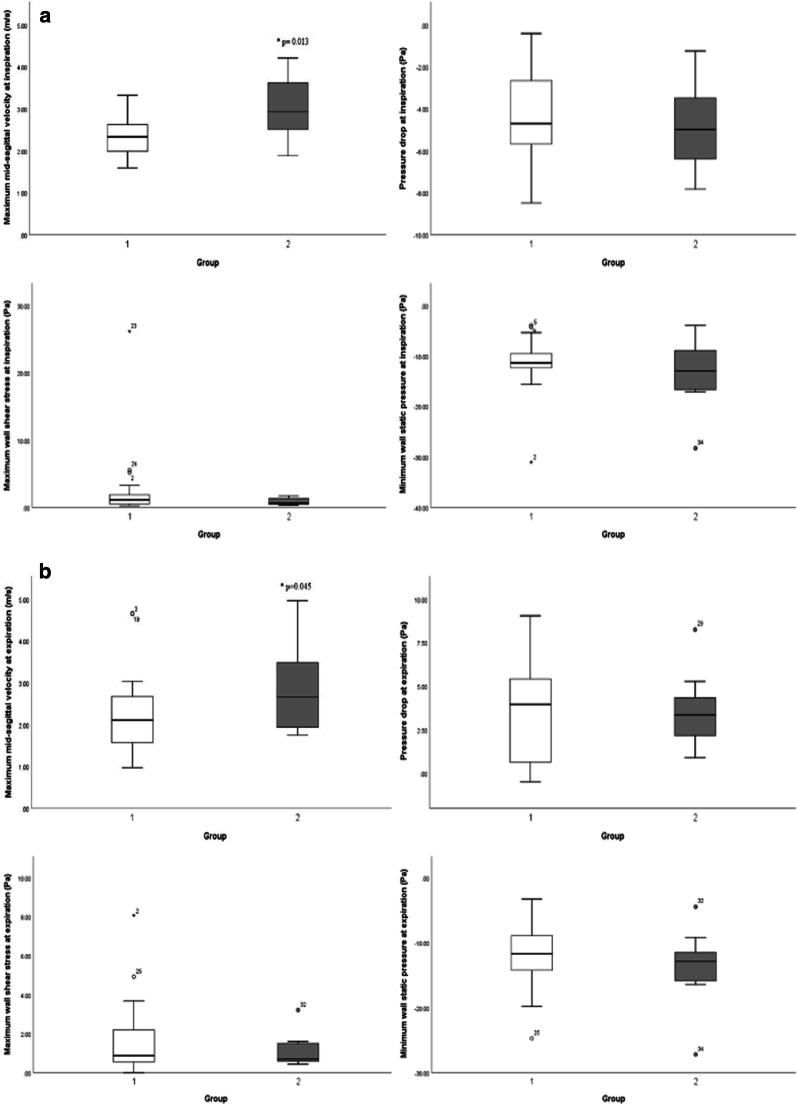
Comparison between the two groups in terms of four aerodynamic parameters at inspiration (**a**) and expiration (**b**)

**Fig. 3:**
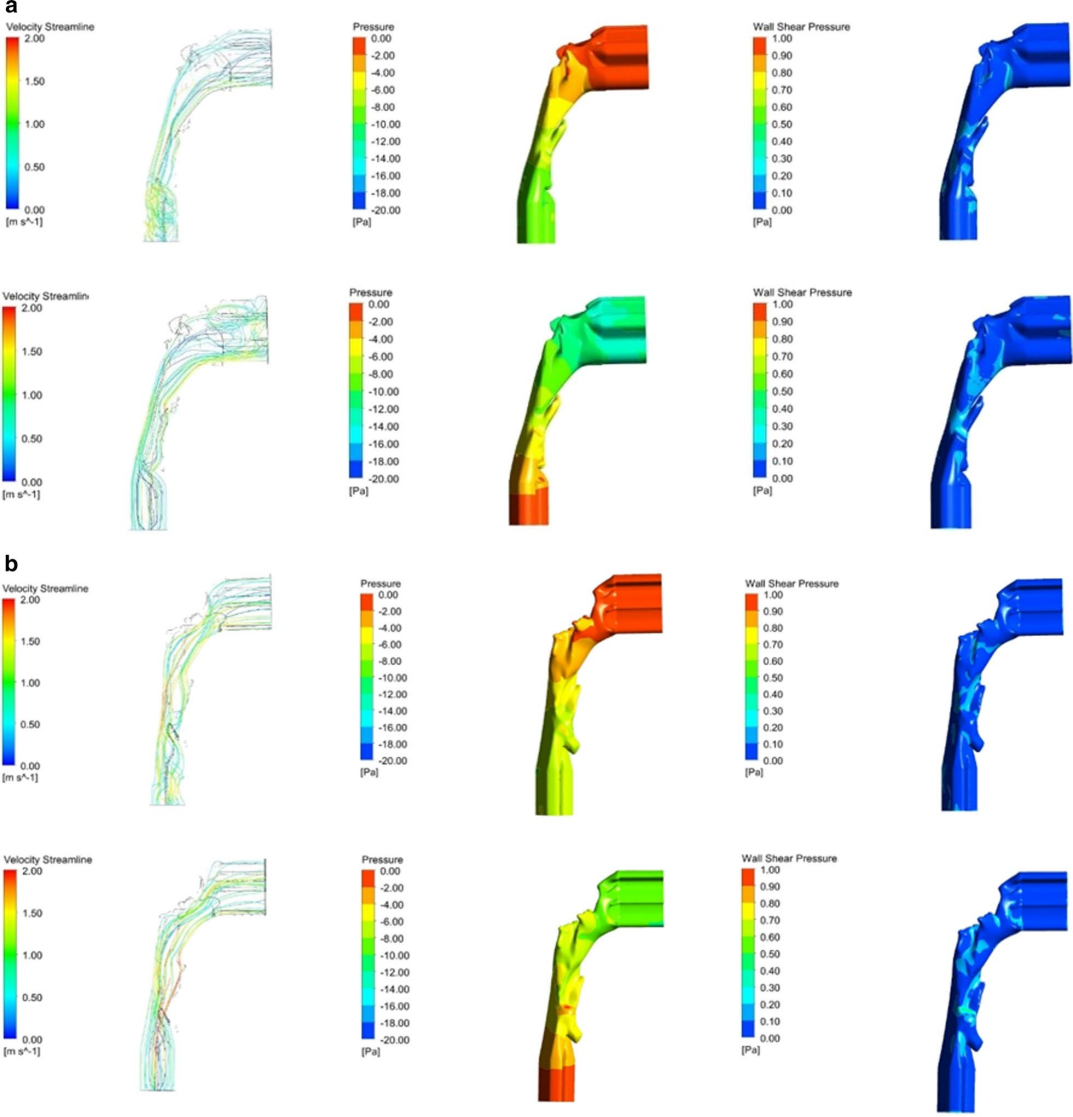
Illustration of the airflow feature in two typical cases with an AN ratio of 0.40 (**a**) and 0.73 (**b**), during inspiration (the up images) and expiration (the down images), respectively. In the case of an AN ratio of 0.40, the ΔP, Maximum V_ms_, Maximum P_ws_ and Minimum P_w_ is − 7.17, 1.95, 0.39, and − 10.75 at inspiration and 7.50, 1.62, 1.44, and − 14.97 at expiration. However, in the case of an AN ratio of 0.73, the corresponding values are − 3.48, 3.62, 1.41, and − 17.06 at inspiration and 5.29, 1.94, 0.59, and − 12.88 at expiration

The mean age of cases was 12.03 ± 1.42 (13 females, 22 males). AN ratios ranged between 0.33 and 0.80 with a mean and standard deviation of 0.54 ± 0.15. We did not find any statistically significant difference between females and males in terms of AN ratio and aerodynamic characteristics. Figure 2 demonstrates the four aerodynamic variables at both inspiration and expiration for the two subgroups. The corresponding descriptive data of aerodynamic parameters for the two groups are listed in Table 2. The maximum V_ms_ in Group 2 exhibits a statistically significant increase of nearly 30% (*p* < 0.05) at both inspiration and expiration in contrast to Group 1. None of the other aerodynamic parameters, including ΔP, maximum wall shear stress (P_ws_), and minimum wall static pressure (P_w_) were significantly different between the two groups at both inspiration and expiration.Fig. 3 illustrates the airflow features of two typical cases with an AN ratio of 0.40 and 0.73, respectively.

**Table 2:** Comparison of the aerodynamic parameters between the two groups defined by the AN ratio 0.6

	Group 1 (n = 25)		Group 2 (n = 10)	Group 1 versus Group 2	
	Mean	SD	Mean	SD	*p* value
*Inspiration*					
ΔP	− 4.38	2.15	− 4.65	2.18	0.373^a^
Maximum V_ms_	2.36	0.46	3.04	0.80	**0.013**^a^
Maximum P_ws_	2.50	5.14	0.93	0.54	0.190^b^
Minimum P_w_	− 11.18	5.09	− 13.14	6.97	0.115^b^
*Expiration*					
ΔP	3.59	2.87	3.60	2.15	0.494^a^
Maximum V_ms_	2.22	0.94	2.92	1.20	**0.045**^b^
Maximum P_ws_	1.64	1.82	1.13	0.84	0.440^b^
Minimum P_w_	− 11.67	4.97	− 13.67	5.87	0.157^a^

Group 1 consisted of the cases with AN ratio less than the 0.6 whereas Group 2 consisted of the cases with AN ratio equal or more than 0.6

^a^Independent samples T test (one-tailed)

^b^Mann–Whitney U test (one-tailed)

Regarding the measurement precision, intra- and interobserver agreement ranged between 0.872 and 0.997 for various aerodynamic parameters.

## Discussion

The present study bridges UA morphology presented as AN ratio and UA function in terms of aerodynamic characteristics by applying CFD simulation. Among all the tested aerodynamic parameters, maximum V_ms_ might be the most sensitive aerodynamic parameter that had demonstrated a significant difference between the two groups [29]. CFD simulation may, therefore, be an additional diagnostic tool to reveal the aerodynamic characteristics within the UA, making the airflow passing through the UA visible. The relationship between UA’s morphology and aerodynamics can be explained by the Bernoulli effect [30], which states that when a fluid flowing through a narrowing region of a tube, an increase in the speed of the fluid coincides with a decrease in pressure. Based on our results, the aerodynamic characteristics of the maximum V_ms_ significantly increased at both inspiration and expiration in cases with an AN ratio of more than 0.6. This finding may imply that clinicians could initially estimate airflow alteration in UA by measuring the AN ratio in a lateral cephalogram. However, caution is needed in extrapolating our results due to the relatively low number of patients, particularly in Group 2. The statistical efficiency would have been higher if there had been an equal number of patients in the two groups.

Besides patients having enlarged adenoid, other conditions such as enlarged tonsils, patients undergo mandibular setback surgery, and obstructive sleep apnea syndrome (OSAS) patients may also cause UA aerodynamic insufficiency. OSAS patients had been mostly studied in previous CFD studies showing significantly different aerodynamic characteristics in UA compared to the healthy subjects. Chen et al. [31] reported that patients with OSAS had a higher airflow resistance at expiration than the control subjects. Wakayama et al. reported that the OSAS patients having nasal obstruction showed a higher maximum velocity and pressure drop at inspiration than the controls [32].

As compared to medical CT, CBCT is more cost-effective and has demonstrated a lower radiation dose [33]. Nevertheless, we must keep in mind that CBCT scans have higher radiation doses than conventional 2D images [34]. In the current study, we utilised the readily available imaging material and applied CFD simulation to investigate the airflow characteristics of these specific groups of cases. It keeps in line with the statement by the American Association of Orthodontists that “the airway and surrounding structures, specifically the adenoids in children, should be evaluated if radiographic records are taken for orthodontic purposes” [35]. Lateral cephalograms are the most commonly performed radiographic examination, which is usually readily available among patients who undergo orthodontic treatment. From the perspective of morphological changes, the AN ratio on a lateral cephalogram may be applied as a useful screening method for estimating the nasopharyngeal volume [17]. The present study reinforces the impact of the AN ratio by its association with airflow features. As the AH may lead to maxillofacial dysmorphisms, multidisciplinary collaboration between orthodontists and otolaryngologists is the key to successful treatment for each individual [36], 37. Based on our result, when the AN ratio is more than 0.6, a noticeable increase in airflow velocity (30%) is observed. Consequently, we may speculate alterations in breathing habits to overcome the nasal obstruction for this group of cases, such as mouth breathing.

Owing to the retrospective study design, a limited number of cases fulfilled the inclusion criteria of having both lateral cephalogram and CBCT among the orthodontic patients. Further prospective research on aerodynamics characteristics involving more cases having enlarged adenoids is warranted. The application of CFD in dentistry is still in the exploration stage of scientific research. One reason is the requirement of 3D imaging to generate a 3D model of UA, which entails automatically higher patient dose as compared to conventional lateral cephalograms. In addition, performing CFD on a large number of cases is challenging since it is highly dependent on the skillfulness of the operator, and the simulation procedure is complicated and time-consuming. CBCT-based aerodynamics simulation is partially performed manually, which may lead to inconsistent reliability for inexperienced operators. Future studies should investigate UA examination using low dose CBCT and the association between UA morphology, respiratory function, and clinical symptoms to better manage children with AH.

## Conclusion

The maximum V_ms_ seems to be the most sensitive aerodynamic parameter for the groups of cases. An AN ratio of more than 0.6 measured on a lateral cephalogram may associate with a noticeably increased maximum V_ms_, which could assist clinicians in estimating the airflow features in the UA.

## Data Availability

All data used and/or analysed during the current study are available from the corresponding author on reasonable request.
